# Clinical outcomes of PFNA vs. long-stem hemiarthroplasty for unstable proximal femur fractures in older adults: a retrospective cohort study

**DOI:** 10.3389/fsurg.2026.1889099

**Published:** 2026-10-05

**Authors:** Peiran Wei, Weihang Zhu, Yan Xu, Xuxiang Li, Zhicheng Cao, Liming Wang, Cheng Tang

**Affiliations:** Department of Orthopaedics, Nanjing First Hospital, Nanjing Medical University, Nanjing, Jiangsu, China

**Keywords:** hemiarthroplasty, older adults, proximal femoral nail antirotation, retrospective study, unstable proximal femur fractures

## Abstract

**Objective:**

The optimal surgical management of unstable extracapsular proximal femur fractures (AO/OTA types A2 and A3) in elderly patients with osteoporosis remains controversial. This study compared clinical outcomes of proximal femoral nail antirotation (PFNA) vs. primary uncemented bipolar hemiarthroplasty with long femoral stem in patients aged ≥75 years. The primary outcome was reoperation rate within 24 months; secondary outcomes included perioperative parameters, functional recovery, complications and mortality.

**Methods:**

A retrospective comparative study was conducted including 174 patients (mean age 82.2 ± 7.1 years, 61.5% female) treated between January 2018 and December 2023. Patients underwent either PFNA (*n* = 96) or hemiarthroplasty (HA, *n* = 78). Primary outcomes included perioperative parameters, functional recovery [Harris Hip Score (HHS), Barthel Index], complications, reoperations, and mortality at 12 and 24 months.

**Results:**

Mean operative time was shorter in the PFNA group (59.6 vs. 74.2 min, *P* < 0.001), while HA patients achieved earlier ambulation (2.8 vs. 6.2 days, *P* < 0.001) and full weight-bearing (4.2 vs. 62.8 days, *P* < 0.001). HHS was higher in the HA group at 3 months (71.6 vs. 66.8, *P* < 0.001) and 6 months (78.2 vs. 74.6, *P* = 0.004), but similar at 24 months. Reoperation rates (7.7% vs. 19.8%, *P* = 0.03) and 2-year mortality (2.6% vs. 11.5%, *P* = 0.02) were significantly lower in the HA group. Multivariable analysis identified PFNA as an independent risk factor for complications (OR = 1.92) and reoperation (sHR = 2.78).

**Conclusions:**

In geriatric patients with unstable extracapsular femoral fractures, long-stem hemiarthroplasty was associated with faster early recovery, lower reoperation rates, and a lower observed 2-year mortality compared to PFNA, which may be considered as an alternative primary option in appropriately selected elderly patients. However, given the retrospective design and potential for residual confounding, this observed association should not be interpreted as a causal effect, which should be confirmed by future prospective randomized trials.

## 1 Introduction

The management of unstable extracapsular proximal femur fractures in the elderly population remains one of the most challenging and debated topics in orthopedic trauma surgery. With the global aging demographic trend, the incidence of these fractures has risen substantially, and this pattern is expected to accelerate in the coming decades. Extracapsular proximal femur fractures predominantly occur in the geriatric population, with most cases resulting from low-energy falls in patients with underlying osteoporosis (1). These fractures are associated with significant morbidity, mortality, and healthcare expenditures, making the optimization of treatment strategies a critical clinical priority (2).

However, despite advances in fixation design and surgical techniques, the failure rate of these unstable fracture patterns remains concerningly steep. A growing body of evidence has demonstrated that AO/OTA type A2 and A3 fractures are associated with significantly increased risk of mechanical complications including cut-out, varus collapse, screw migration, and nonunion (3). In a cohort study of 97 trochanteric fractures treated with proximal femoral locking plates, He et al. reported a failure rate of 50% in unstable fractures compared to only 17% in stable patterns, with patients over 60 years of age suffering from unstable fractures experiencing a failure rate as high as 60.5% (4). Similarly, comparative studies of intramedullary devices have documented elevated rates of complications and reoperations in unstable fracture types, particularly in elderly patients with compromised bone quality.

The presence of osteoporosis compounds these challenges by diminishing the purchase of implants in osteoporotic bone, thereby increasing the susceptibility to fixation failure (5). Elderly patients with osteoporosis exhibit reduced bone mineral density, thinning of the cortical bone, and diminished trabecular microarchitecture, all of which compromise the mechanical integrity of the bone-implant construct (6). When osteoporosis coexists with unstable fracture patterns, the combination creates a “perfect storm” for internal fixation failure, as the already tenuous mechanical stability is greatly undermined by poor bone quality (6, 7). Perhaps even more consequential than the mechanical failure itself is the clinical sequelae that follow uncertain internal fixation, typically requiring prolonged periods of restricted weight-bearing or bed rest to protect the fragile construct during the healing process. This enforced immobilization in an already vulnerable geriatric population substantially increases the risk of devastating complications including deep vein thrombosis, pulmonary embolism, pressure ulcers, pneumonia, and generalized deconditioning (7). These complications contribute directly to the elevated mortality rates observed after hip fractures, with studies documenting 1-year mortality approaching 20%–30% in elderly patients (8). The inability to mobilize early after internal fixation of unstable fractures thus represents a critical restriction of this treatment approach.

In response to these challenges, some investigators have proposed primary hemiarthroplasty as an alternative treatment strategy for unstable extracapsular proximal femur fractures in selected elderly patients (9). Proponents argue that arthroplasty offers the distinct advantage of immediate postoperative weight-bearing capacity, thereby circumventing the period of protected weight-bearing required after internal fixation and potentially reducing the risks associated with prolonged recumbency (10). Several retrospective series have reported favorable outcomes with hemiarthroplasty, including early mobilization, acceptable functional results, and acceptable complication profiles (10–12). Despite the accumulating evidence, the optimal surgical management of unstable extracapsular proximal femur fractures in elderly patients with osteoporosis remains controversial, and high-quality comparative studies are relatively scarce. The majority of existing literature consists of limited case series or comparisons that do not adequately control for fracture morphology, bone quality, and patient comorbidities. Furthermore, numerous studies combine stable and unstable fracture patterns, potentially obscuring crucial differences in treatment outcomes between these distinct entities (13–15). There is a clear need for well-designed comparative studies focusing specifically on unstable extracapsular proximal femur fractures in the osteoporotic elderly population, with comprehensive assessment of both early perioperative outcomes and longer-term functional recovery.

## 2 Materials and methods

### 2.1 Study design and patient selection

A retrospective comparative study was conducted at Nanjing First Hospital between January 2018 and December 2023. After obtaining approval from the institutional review board, we reviewed the medical records of all patients who underwent surgical treatment for extracapsular proximal femur fractures. The requirement for informed consent was waived due to the retrospective nature of the study.

A total of 347 consecutive patients with extracapsular proximal femur fractures were initially identified from our hospital's electronic medical record system. An independent investigator reviewed all medical records and imaging studies. Inclusion Criteria were as follows: age ≥75 years at the time of surgery; diagnosed with unstable extracapsular proximal femur fracture classified as AO/OTA type A2 or A3; underwent either proximal femoral nail antirotation (PFNA) internal fixation or primary uncemented bipolar hemiarthroplasty with long femoral stem (Chunlizhengda, China); complete medical records including preoperative, perioperative, and postoperative data with minimum 24-month follow-up data available. Loss to follow-up was defined as absence of contact at any scheduled follow-up time point (3, 6, 12, and 24 months postoperatively). Death was not classified as loss to follow-up; mortality data were captured through medical records and telephone interviews with family members.

Exclusion criteria were pathological fractures due to primary or metastatic tumors (*n* = 12); polytrauma patients with Injury Severity Score > 16 (*n* = 8); previous hip surgery or ipsilateral lower extremity fractures (*n* = 15); active infection at the time of surgery (*n* = 3); severe cognitive impairment precluding functional assessment (Mini-Mental State Examination Score < 10) (*n* = 22); high-energy trauma mechanisms (traffic accidents, falls from height, et al.) (*n* = 14); stable fracture patterns (AO/OTA type A1) (*n* = 68); incomplete medical records or loss to follow-up within 24 months (*n* = 31).

### 2.2 Final study cohort and surgical procedures

After applying the inclusion and exclusion criteria, a total of 174 patients were included in the final analysis. Given the retrospective nature of this study, a formal *a priori* sample size calculation was not performed. The sample size was determined by the number of eligible patients treated at our institution during the study period. A *post-hoc* precision analysis indicated that the achieved sample size (*n* = 174) provided sufficient precision for the primary outcome (reoperation rate difference between groups: 12.1% absolute difference, 95% CI: 4.3–19.9). Based on the surgical procedure received, patients were divided into the PFNA Group (*n* = 96) and the Hemiarthroplasty Group (HA Group, *n* = 78). The allocation to treatment groups was determined by the attending surgeon's preference, patient and family discussion, and clinical circumstances. Flowchart of patient screening and allocation were presented in Figure 1.

**Figure 1:**
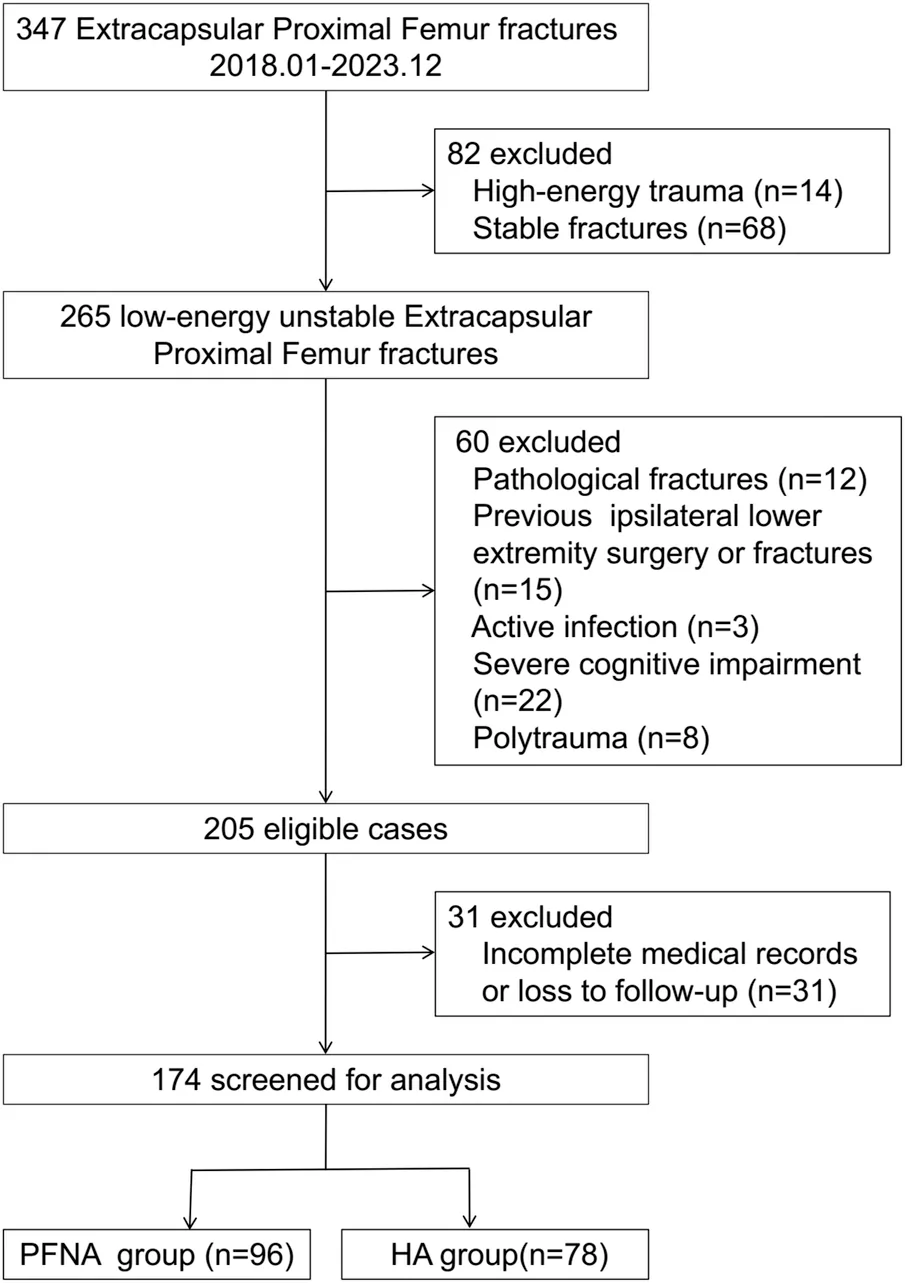
Flowchart of patient screening of hemiarthroplasty and PFNA internal fixation for unstable extracapsular proximal femur fractures.

In the PFNA group, all patients underwent surgery using the Proximal Femoral Nail Antirotation (PFNA, Waston, China) system. Patients were positioned supine on a fracture table under general or spinal anesthesia. Closed reduction was attempted under fluoroscopic guidance, with the goal of achieving optimal anatomical or slight valgus reduction (neck-shaft angle 130°–135°). The entry point was at the tip of the greater trochanter, and the intramedullary nail was inserted without reaming. A helical blade of appropriate length was inserted and locked under fluoroscopic guidance, with the blade positioned in the inferior-central or central portion of the femoral head to minimize cut-out risk. Distal locking was performed using a static screw. All PFNA procedures were performed by one group of experienced surgeons following standardized institutional protocols. Postoperatively, patients were instructed to maintain toe-touch weight-bearing for 6–8 weeks, progressing to full weight-bearing based on radiographic evidence of healing. As in such fracture patterns with compromised bone quality, early full weight-bearing may concentrate excessive stress on the implant, increasing the risk of fixation failure (16, 17). Therefore, the weight-bearing restriction was therefore instituted to protect the bone-implant construct during the healing phase.

In the HA group, all patients underwent uncemented bipolar hemiarthroplasty using a long femoral stem (Chunlizhengda, China). The procedure was performed through a direct lateral approach. Patients were positioned in the lateral decubitus position under general or spinal anesthesia. The fractured femoral head and neck were resected. After sequential reaming and broaching, an uncemented long stem (tapered, grit-blasted design; diameters 11–18 mm, lengths 200 mm) was implanted to achieve distal diaphyseal fixation beyond the fracture site. The stem was sized according to preoperative templating, with the goal of obtaining cortical engagement in the diaphysis while bypassing the comminuted metaphysis. The greater trochanter and associated comminuted fragments were reconstructed using 2.0 mm cerclage wires in a figure-of-eight configuration to restore abductor mechanism function. The lesser trochanter was repaired when the fragment was large enough to accommodate a wire. A bipolar head of appropriate size was assembled, and the hip was reduced. No intraoperative femoral fractures occurred in all cases. All procedures were performed by one group of experienced senior surgeons following standardized protocols. Postoperatively, patients were allowed full weight-bearing as tolerated with a walker from the first postoperative day. Both group patients received anticoagulation treatment for 35 days after the operation.

### 2.3 Data collection and outcome measures

Independent investigators collected the following demographic and clinical characteristics for all patients by reviewing electronic medical records, anesthesia records, nursing notes, outpatient follow-up documentation, and functional and complication measures.

Operative time, intraoperative blood loss, intraoperative and postoperative blood transfusion requirements, postoperative drainage volume (mL, recorded at 24–48 h postoperatively), and total length of hospital stay were also recorded. Time to first ambulation (days postoperatively, defined as the first day the patient was able to walk at least 5 meters with assistance) and time to full weight-bearing (days postoperatively, defined as the day the patient could bear full weight without assistance or walking aid) were documented for all patients from nursing and physical therapy records. Radiographic assessments for HA patients were performed independently by two orthopedic surgeons and included evaluation of stem alignment, fracture healing, trochanteric union, and prosthetic loosening or subsidence. Disagreements were resolved by consensus to ensure reliability.

Functional assessment was performed using the Harris Hip Score (HHS) preoperatively and at 3, 6, 12, and 24 months postoperatively. Preoperative HHS was based on the patient's recalled pre-fracture functional status, obtained through structured interview with the patient and, when necessary, with family members at the time of admission. The HHS evaluates pain (44 points), function (47 points), deformity (4 points), and range of motion (5 points), with higher scores indicating better function (excellent: 90–100, good: 80–89, fair: 70–79, poor: <70). At the 12- and 24-month follow-up, we also assessed activities of daily living using the Barthel Index and health-related quality of life using the EuroQol-5D (EQ-5D) questionnaire.

Surgical complications included superficial wound infection (requiring antibiotics only), deep surgical site infection (requiring surgical debridement), hematoma formation, and wound dehiscence. For the PFNA group, implant-specific complications included cut-out of the helical blade (penetration through the femoral head), varus collapse (>10° change in neck-shaft angle compared to immediate postoperative radiographs), nonunion (absence of bridging trabeculae at 6 months), malunion (healing in unacceptable alignment), screw penetration into the joint, femoral shaft fracture at the nail tip, and implant breakage. For the HA group, prosthesis-specific complications included prosthetic dislocation, periprosthetic fracture, aseptic loosening (progressive radiolucent lines ≥2 mm in any DeLee and Charnley zone), prosthetic subsidence (>5 mm migration), and heterotopic ossification (Brooker classification).

Medical complications recorded included deep vein thrombosis (confirmed by Doppler ultrasound in all patients postoperatively), pulmonary embolism (confirmed by CT angiography in symptomatic patients), pneumonia (clinical symptoms with CT confirmation), urinary tract infection (positive urine culture), acute myocardial infarction (ECG changes and cardiac enzyme elevation), cerebrovascular accident (new neurological deficit with CT and MRI confirmation), pressure ulcers, and postoperative delirium (assessed using the Confusion Assessment Method). Re-fractures, reoperations, and mortality were obtained from medical records and follow-up calls. Mortality was assessed at 1- and 2-year postoperatively.

The primary outcome of this study was reoperation rate within 24 months after the initial surgery. Reoperation was defined as any subsequent surgical procedure related to the index fracture, including conversion to arthroplasty, revision internal fixation, debridement for infection, or closed reduction for dislocation. This endpoint was selected *a priori* because it represents the most clinically significant and objectively verifiable measure of treatment failure. Secondary outcomes included perioperative parameters (operative time, blood loss, transfusion, hospital stay), functional recovery (Harris Hip Score, Barthel Index, EQ-5D), postoperative complications (surgical and medical) and mortality.

### 2.4 Statistical analysis

Statistical analysis was performed using SPSS version 26.0 (IBM Corp., Armonk, NY, USA) for Windows. All statistical tests were two-tailed, and a *P*-value <0.05 was considered statistically significant.

Continuous variables were tested for normality using the Shapiro–Wilk test. Normally distributed continuous variables were expressed as mean ± standard deviation (SD). Categorical variables were expressed as frequencies and percentages.

For continuous variables, comparisons between the PFNA and HA groups were performed using the independent samples t-test for normally distributed data or the Mann–Whitney *U* test for non-normally distributed data. For categorical variables, comparisons were performed using the chi-square test or Fisher's exact test (when expected cell counts were <5) as appropriate.

Longitudinal changes in Harris Hip Score (HHS) were analyzed using a linear mixed-effects model with restricted maximum likelihood estimation. The model included fixed effects for group (PFNA vs. HA), time (preoperative, 3, 6, 12, and 24 months), and group-by-time interaction, with a random intercept for each patient to account for within-subject correlation. This approach uses all available measurements without requiring complete case data and appropriately handles missing data under the missing-at-random assumption. Data from patients who died or underwent reoperation were included up to their last available assessment. *Post-hoc* comparisons with Bonferroni correction were performed for significant interaction effects.

For the primary outcome (reoperation rate), the absolute risk difference with 95% confidence interval was calculated. Reoperation-free survival was analyzed using the Kaplan–Meier method with the log-rank test to compare survival distributions between groups. Patients were censored at the time of last follow-up or death. Cumulative survival rates at 12 and 24 months were reported with 95% confidence intervals. For reoperation, cumulative incidence functions were estimated using the Fine-Gray competing-risk model, with death as a competing event. Subdistribution hazard ratios (sHR) with 95% confidence intervals were reported.

To identify independent risk factors for complications, multivariable logistic regression analysis was performed using the Wald test. Variables with *P* < 0.10 in univariable analysis were entered into the multivariable model using backward stepwise selection. Results were reported as odds ratios (OR) with 95% confidence intervals (CI). Model calibration was assessed using the Hosmer-Lemeshow test, and discriminative ability was evaluated using the AUC.

For the reoperation model, given the limited number of events and the potential for overfitting, we prespecified a reduced set of three clinically relevant predictors: surgical procedure (PFNA vs. HA), AO/OTA fracture type (A3 vs. A2), and osteoporosis (*T*-score ≤ −2.5). To account for death as a competing event, we applied competing-risk regression according to the method of Fine and Gray, reporting subdistribution hazard ratios (sHR) with 95% CIs. Bootstrap internal validation with 1,000 resamples was performed to assess model stability and optimism-corrected performance.

Continuous variables, including age, Charlson Comorbidity Index (CCI), and bone mineral density *T*-score, were dichotomized for multivariable analysis based on clinically meaningful thresholds. Age was dichotomized at 85 years, consistent with the commonly researched in geriatric research (18). CCI was dichotomized at >5, reflecting a high comorbidity burden associated with increased postoperative risks in hip fracture populations (19). *T*-score was dichotomized at ≤−2.5, based on the WHO diagnostic criteria for osteoporosis (20). These cutoffs were predefined prior to analysis, not data-driven.

For secondary outcomes, point estimates with 95% confidence intervals were reported alongside *P* values. No adjustment for multiple comparisons was made, as the primary/secondary distinction was clearly prespecified and secondary analyzes were considered exploratory.

To assess the potential impact of loss to follow-up on the primary outcomes, a best-case/worst-case sensitivity analysis was performed for mortality and reoperation. In the best-case scenario, all patients lost to follow-up in the PFNA group were assumed to have favorable outcomes (alive for mortality analysis; no reoperation for reoperation analysis), while all those lost in the HA group were assumed to have unfavorable outcomes (dead; reoperation). The worst-case scenario applied the opposite assumptions. The direction and magnitude of effect were compared across scenarios to evaluate the robustness of the main findings.

## 3 Results

### 3.1 Demographic and clinical characteristics

A total of 174 patients with unstable extracapsular proximal femur fractures (AO/OTA types A2 and A3) who met the inclusion criteria were included in this retrospective study. Among them, 96 patients (55.2%) underwent proximal femoral nail antirotation (PFNA) internal fixation, and 78 patients (44.8%) underwent uncemented bipolar hemiarthroplasty with long femoral stem (HA group). Among the 174 included patients, 31 (17.8%) were lost to follow-up within 24 months: 17 (17.7%) in the PFNA group and 14 (17.9%) in the HA group (*P* = 0.98). Reasons for loss to follow-up included refusal to return for scheduled visits (*n* = 18), relocation to other cities (*n* = 8), and inability to contact (*n* = 5). All deaths were captured and included in the analysis (PFNA: *n* = 11; HA: *n* = 2); no death was excluded as loss to follow-up.

The demographic and clinical characteristics of both groups are summarized in Table 1 and Supplementary Table S1. There were no statistically significant differences between the two groups in any of the demographic and clinical parameters, indicating successful comparability of the cohorts. The mean age was 82.9 ± 6.9 years in the PFNA group and 81.5 ± 7.4 years in the HA group (*P* = 0.58). Both groups had a predominance of female patients (PFNA: 62.5%, HA: 60.3%; *P* = 0.65). The mean body mass index was similar between groups (PFNA: 23.6 ± 3.2 kg/m^2^, HA: 23.3 ± 3.5 kg/m^2^; *P* = 0.55). Regarding fracture characteristics, the distribution of AO/OTA fracture types was comparable between groups: type A2 fractures accounted for 63.5% (*n* = 61) in the PFNA group and 61.5% (*n* = 48) in the HA group, while type A3 fractures comprised 36.5% (*n* = 35) and 38.5% (*n* = 30), respectively (*P* = 0.68). The prevalence of osteoporosis was high in both groups (PFNA: 84.4%, HA: 87.2%; *P* = 0.60), with mean bone mineral density *T*-scores of −3.3 ± 0.8 and −3.4 ± 0.7, respectively (*P* = 0.38). The mean time from injury to surgery was 3.6 ± 1.9 days in the PFNA group and 3.8 ± 2.2 days in the HA group (*P* = 0.52). Pre-fracture walking ability was also comparable between groups (*P* = 0.74).

**Table 1:** Demographic and clinical characteristics of patients.

Characteristic	PFNA group (*n* = 96)	HA group (*n* = 78)	*P*-value
Demographics			
Age (years, mean ± SD)	82.9 ± 6.9	81.5 ± 7.4	0.58*
Age distribution (*n*, %)			0.72**
75–80 years	32 (33.3%)	24 (30.8%)	
80–85 years	48 (50.0%)	40 (51.3%)	
≥85 years	16 (16.7%)	14 (17.9%)	
Gender (*n*, %)			0.65**
Male	36 (37.5%)	31 (39.7%)	
Female	60 (62.5%)	47 (60.3%)	
Body mass index (kg/m^2^, mean ± SD)	23.6 ± 3.2	23.3 ± 3.5	0.55*
Fracture side (*n*, %)			0.82**
Left	51 (53.1%)	40 (51.3%)	
Right	45 (46.9%)	38 (48.7%)	
AO/OTA fracture type (*n*, %)			0.68**
A2 (multi-fragmentary)	61 (63.5%)	48 (61.5%)	
A3 (reverse obliquity/transverse)	35 (36.5%)	30 (38.5%)	
Bone mineral density *T*-score (mean ± SD)	−3.3 ± 0.8	−3.4 ± 0.7	0.38*
Osteoporosis (*T*-score ≤ −2.5) (*n*, %)	81 (84.4%)	68 (87.2%)	0.60**
Time from injury to surgery (days, mean ± SD)	3.6 ± 1.9	3.8 ± 2.2	0.52*
Pre-fracture walking ability (*n*, %)			0.74**
Independent without aid	28 (29.2%)	21 (26.9%)	
Independent with cane	44 (45.8%)	38 (48.7%)	
Walker-dependent	24 (25.0%)	19 (24.4%)	
Comorbidity indices			
Charlson Comorbidity Index (CCI, mean ± SD)	5.3 ± 1.9	5.5 ± 2.0	0.50*
ASA classification (*n*, %)			0.68**
Grade II	21 (21.9%)	16 (20.5%)	
Grade III	62 (64.6%)	53 (68.0%)	
Grade IV	13 (13.5%)	9 (11.5%)	

PFNA, proximal femoral nail antirotation; HA, hemiarthroplasty; SD, standard deviation; AO/OTA, Arbeitsgemeinschaft für Osteosynthesefragen/Orthopaedic Trauma Association; ASA, American Society of Anesthesiologists.

* Independent samples *t*-test.

** Chi-square test.

Table 1 also presents the detailed preoperative comorbidities and cognitive function assessment. The Charlson Comorbidity Index (CCI) was similar between groups (PFNA: 5.3 ± 1.9, HA: 5.5 ± 2.0; *P* = 0.50). The distribution of ASA classification was also comparable (*P* = 0.68), with the majority of patients in both groups classified as ASA grade III (PFNA: 64.6%, HA: 68.0%). The prevalence of specific comorbidities did not differ significantly between groups, such as hypertension (PFNA: 70.8%, HA: 73.1%; *P* = 0.74), diabetes mellitus (PFNA: 42.7%, HA: 41.0%; *P* = 0.82), coronary artery disease (PFNA: 34.4%, HA: 35.9%; *P* = 0.83), chronic obstructive pulmonary disease (PFNA: 22.9%, HA: 21.8%; *P* = 0.86), et al. Antiplatelet or anticoagulant medication use was reported in 49.0% of PFNA patients and 52.6% of HA patients (*P* = 0.64). Preoperative hemoglobin (PFNA: 11.7 ± 1.7 g/dL, HA: 11.5 ± 1.9 g/dL; *P* = 0.46) was comparable between groups.

Cognitive function assessment revealed similar mean MMSE scores (PFNA: 23.8 ± 4.3, HA: 23.4 ± 4.6; *P* = 0.55). The distribution of cognitive status was comparable (*P* = 0.62), with normal cognition (MMSE ≥ 25) in 54.2% of PFNA patients and 51.3% of HA patients, mild impairment (MMSE 21–24) in 34.4% and 35.9%, respectively, and moderate impairment (MMSE 10–20) in 11.5% and 12.8%, respectively. Preoperative delirium was present in 7.3% of PFNA patients and 6.4% of HA patients (*P* = 0.82). The preoperative Barthel Index was also similar between groups (PFNA: 81.6 ± 16.2, HA: 79.8 ± 17.8; *P* = 0.49) (Supplementary Table S1).

### 3.2 Perioperative and functional outcomes

Table 2 summarizes the perioperative parameters and rehabilitation outcomes. The mean operative time was shorter in the PFNA group compared to the HA group (59.6 ± 16.4 vs. 74.2 ± 19.8 min; *P* < 0.001). Intraoperative blood loss was comparable between the two groups (232.8 ± 52.6 vs. 248.4 ± 92.3 mL; *P* = 0.45), and the intraoperative blood transfusion rate was also similar (*P* = 0.45). The total length of hospital stay was similar between groups, with a mean of 10.2 ± 4.6 days in the PFNA group and 10.8 ± 5.3 days in the HA group (*P* = 0.42). Patients in the HA group achieved first ambulation significantly earlier than those in the PFNA group (2.8 ± 1.3 vs. 6.2 ± 2.6 days; *P* < 0.001). More strikingly, the time to full weight-bearing was dramatically shorter in the HA group (4.2 ± 2.4 days) compared to the PFNA group (62.8 ± 14.2 days; *P* < 0.001), reflecting the immediate postoperative weight-bearing protocol for arthroplasty patients vs. the prolonged protected weight-bearing required after internal fixation.

**Table 2:** Perioperative and postoperative outcome comparison.

Outcome measure	PFNA group (*n* = 96)	HA group (*n* = 78)	*P*-value
Perioperative parameters			
Operative time (min, mean ± SD)	59.6 ± 16.4	74.2 ± 19.8	**<0.001** *
Intraoperative blood loss (mL, mean ± SD)	232.8 ± 52.6	248.4 ± 92.3	0.45*
Intraoperative blood transfusion (*n*, %)	49 (51.0%)	45 (57.7%)	0.45**
Length of hospital stay (days, mean ± SD)	10.2 ± 4.6	10.8 ± 5.3	0.42*
Rehabilitation parameters			
Time to first ambulation (days, mean ± SD)	6.2 ± 2.6	2.8 ± 1.3	**<0.001** *
Time to full weight-bearing (days, mean ± SD)	62.8 ± 14.2	4.2 ± 2.4	**<0.001** *
Functional outcomes			
Harris Hip Score at 3 months (mean ± SD)	66.8 ± 9.2	71.6 ± 8.4	**<0.001** *
Harris Hip Score at 6 months (mean ± SD)	74.6 ± 8.4	78.2 ± 7.8	**0.004** *
Harris Hip Score at 12 months (mean ± SD)	80.4 ± 7.2	82.6 ± 6.8	0.08*
Harris Hip Score at 24 months (mean ± SD)	81.8 ± 6.6	82.4 ± 6.9	0.56*
Barthel Index at 12 months (mean ± SD)	77.4 ± 13.2	81.8 ± 12.4	**0.03** *
Barthel Index at 24 months (mean ± SD)	79.6 ± 15.8	84.3 ± 14.5	**0.04** *
EQ-5D score at 12 months (mean ± SD)	0.71 ± 0.15	0.74 ± 0.14	0.18*
EQ-5D score at 24 months (mean ± SD)	0.75 ± 0.17	0.78 ± 0.12	0.19*

Bold values indicate statistically significant differences (*P* < 0.05). PFNA, proximal femoral nail antirotation; HA, hemiarthroplasty; SD, standard deviation; EQ-5D, EuroQol-5D.

* Independent samples *t*-test.

** Chi-square test.

The HHS was assessed preoperatively and at multiple time points postoperatively. At 3 months postoperatively, the HA group demonstrated significantly higher HHS compared to the PFNA group (71.6 ± 8.4 vs. 66.8 ± 9.2; *P* < 0.001). This significant difference persisted at 6 months (HA: 78.2 ± 7.8, PFNA: 74.6 ± 8.4; *P* = 0.004). However, by 12 months postoperatively, the difference between groups narrowed and was no longer statistically significant (HA: 82.6 ± 6.8, PFNA: 80.4 ± 7.2; *P* = 0.08). At 24 months, both groups achieved similar HHS (HA: 82.4 ± 6.9, PFNA: 81.8 ± 6.6; *P* = 0.56). The linear mixed-effects model demonstrated a significant main effect of time (*P* < 0.001), indicating progressive improvement in both groups. There was also a significant group-by-time interaction (*P* < 0.001), confirming that the trajectory of functional recovery differed between groups, with the HA group achieving faster early recovery but similar outcomes at 12 and 24 months. The number of patients assessed at each time point is shown in Supplementary Table S5.

At 12 months postoperatively, the Barthel Index was significantly higher in the HA group compared to the PFNA group (81.8 ± 12.4 vs. 77.4 ± 13.2, *P* = 0.03); at 24 months, the HA group also showed better scores (84.3 ± 14.5 vs. 79.6 ± 15.8, *P* = 0.04), indicating better activities of daily living performance. The EQ-5D score at 12 and 24 months was also higher in the HA group, but this difference did not reach statistical significance (0.74 ± 0.14 vs. 0.71 ± 0.15, *P* = 0.18; 0.78 ± 0.12 vs. 0.75 ± 0.17, *P* = 0.19).

### 3.3 Complications, reoperations and mortality

As presented in Table 3, the overall complication rate was 39.6% (*n* = 38) in the PFNA group and 35.9% (*n* = 28) in the HA group, with no statistically significant difference between groups (*P* = 0.62). Superficial wound infection occurred in 4.2% (*n* = 4) of PFNA patients and 6.4% (*n* = 5) of HA patients (*P* = 0.73), and one deep surgical site infection was observed in each group. Deep vein thrombosis occurred in 14.6% (*n* = 14) of PFNA patients and 3.8% (*n* = 3) of HA patients (*P* = 0.02). Pulmonary embolism was rare, occurring in 2.1% (*n* = 2) and 1.3% (*n* = 1) of patients in the PFNA and HA groups, respectively (*P* = 1.00). Pneumonia was observed in 8.3% (*n* = 8) of PFNA patients and 6.4% (*n* = 5) of HA patients (*P* = 0.63). Urinary tract infection occurred in 4.1% (*n* = 4) and 5.1% (*n* = 4) of patients in the PFNA and HA groups, respectively (*P* = 0.68). Cardiovascular events included acute myocardial infarction in 3.1% (*n* = 3) of PFNA patients and 5.1% (*n* = 4) of HA patients (*P* = 0.70), and cerebrovascular accident in 2.1% (*n* = 2) and 1.3% (*n* = 1) of patients, respectively (*P* = 0.66). Pressure ulcers were more common in the PFNA group (6.3%, *n* = 6) compared to the HA group (2.6%, *n* = 2), although this difference did not reach statistical significance (*P* = 0.30). Postoperative delirium occurred in 11.5% (*n* = 11) of PFNA patients and 10.3% (*n* = 8) of HA patients (*P* = 0.80).

**Table 3:** Complications, reoperations and mortality.

Outcome measure	PFNA group (*n* = 96)	HA group (*n* = 78)	*P*-value
Overall complications (*n*, %)	38 (39.6%)	28 (35.9%)	0.62*
Superficial wound infection	4 (4.2%)	5 (6.4%)	0.73**
Deep surgical site infection	1 (1.0%)	1 (1.3%)	1.0**
Deep vein thrombosis	14 (14.6%)	3 (3.8%)	**0.02**
Pulmonary embolism	2 (2.1%)	1 (1.3%)	1.00**
Pneumonia	8 (8.3%)	5 (6.4%)	0.63*
Urinary tract infection	4 (4.1%)	4 (5.1%)	0.68*
Acute myocardial infarction	3 (3.1%)	4 (5.1%)	0.70**
Cerebrovascular accident	2 (2.1%)	1 (1.3%)	0.66**
Pressure ulcers	6 (6.3%)	2 (2.6%)	0.30**
Delirium (postoperative)	11 (11.5%)	8 (10.3%)	0.80*
Refracture	3 (3.1%)	3 (3.8%)	0.80*
Reoperations (*n*, %)	19 (19.8%)	6 (7.7%)	**0.03** *
Conversion to arthroplasty	14 (14.6%)	N/A	
Revision internal fixation	3 (3.1%)	2 (2.6%)	
Debridement/irrigation	2 (2.1%)	1 (1.3%)	
Closed reduction for dislocation	N/A	1 (1.3%)	
Revision arthroplasty	N/A	1 (1.3%)	
Revision arthroplasty and fixation	N/A	1 (1.3%)	
Mortality			
1-year mortality	8 (8.3%)	1 (1.3%)	**0.04** *
2-year mortality	11 (11.5%)	2 (2.6%)	**0.02** *
Cause of death (*n*)			
Pneumonia	3	1	
Cardiovascular events	2	1	
Multi-organ failure	2	1	
Other causes	2	1	
Timing of death (*n*)			
≤1month	2 (2.1%)	0 (0%)	
1–6 months	4 (4.2%)	1 (1.3%)	
7–12 months	2 (2.1%)	0 (0%)	
13–24 months	3 (3.1%)	1 (1.3%)	

Bold values indicate statistically significant differences (*P* < 0.05). PFNA, proximal femoral nail antirotation; HA, hemiarthroplasty; SD, standard deviation; TAD, tip-apex distance; N/A, not applicable.

* Chi-square test.

** Fisher's exact test.

There were 3 re-fracture cases in each group. And the primary outcome reoperation rate was significantly higher in the PFNA group compared to the HA group (19.8% [19/96] vs. 7.7% [6/78]; absolute risk difference: 12.1%, 95% CI: 4.3–19.9; *P* = 0.03). In the PFNA group, reoperations included conversion to arthroplasty for failed internal fixation or nonunion (*n* = 14, 14.6%), revision internal fixation (*n* = 3, 3.1%), and debridement and irrigation for infection (*n* = 2, 2.1%). In the HA group, reoperations included closed reduction for dislocation (*n* = 1, 1.3%) and debridement and irrigation for deep infection (*n* = 1, 1.3%). Periprosthetic fracture was observed in 3 patients (3.8%), with two patients undergoing internal fixation revision (*n* = 2, 2.6%), one patient undergoing revision arthroplasty and fixation (*n* = 1, 1.3%). In addition, one patient underwent revision arthroplasty for prosthesis loosening (*n* = 1, 1.3%). No patient underwent more than one re-operation during the follow-up period. In the HA group, no trochanteric nonunion or wire/cable breakage was observed during the follow-up period.

The 1-year mortality rate was observed to be lower in the HA group compared to the PFNA group (1.3% vs. 8.3%, *P* = 0.04). The 2-year mortality rate was also observed to be lower in the HA group (2.6% vs. 11.5%, *P* = 0.02). Death occurred predominantly within the first 6 months postoperatively (PFNA: *n* = 6, 6.3%; HA: *n* = 1, 1.3%). The leading causes of death were pneumonia (*n* = 4), cardiovascular events (*n* = 3), multi-organ failure (*n* = 3), and other causes (*n* = 3) (Table 3).

### 3.4 Risk factors for overall complications and reoperation

Multivariable logistic regression analysis identified independent risk factors for overall complications and reoperation. After adjusting for potential confounders, the following factors were independently associated with increased risk of overall complications: age ≥85 years (OR 2.84, 95% CI 1.32–6.11, *P* = 0.008), Charlson Comorbidity Index > 5 (OR 2.56, 95% CI 1.28–5.12, *P* = 0.008), osteoporosis (*T*-score ≤ −2.5) (OR 2.18, 95% CI 1.12–4.24, *P* = 0.02), AO/OTA type A3 fracture (OR 1.96, 95% CI 1.02–3.77, *P* = 0.04), and PFNA internal fixation (compared to HA) (OR 1.92, 95% CI 1.35–2.73, *P* = 0.01) (Table 4, complete regression output, including coefficients, standard errors and Wald statistics, is shown in Supplementary Table S2).

**Table 4:** Independent risk factors for overall complications and reoperation.

Risk factors	Complications (Logistic regression)	Reoperation (Fine-Gray competing-risk regression)
Age ≥ 85 years	OR = 2.84, 95% CI: 1.32–6.11, *P* = 0.008	
Charlson Comorbidity Index > 5	OR = 2.56, 95% CI: 1.28–5.12, *P* = 0.008	
Osteoporosis (*T*-score ≤ −2.5)	OR = 2.18, 95% CI: 1.12–4.24, *P* = 0.02	sHR = 2.84, 95% CI: 1.48–5.45, *P* = 0.002
AO/OTA type A3 fracture	OR = 1.96, 95% CI: 1.02–3.77, *P* = 0.04	sHR = 2.51, 95% CI: 1.02–6.18, *P* = 0.045
PFNA (vs. HA)	OR = 1.92, 95% CI: 1.35–2.73, *P* = 0.01	sHR = 2.78, 95% CI: 1.05–7.36, *P* = 0.04

OR, odds ratio; sHR, subdistribution hazard ratio; CI, confidence interval; HA, hemiarthroplasty; PFNA, proximal femoral nail antirotation. For the reoperation model, results were derived from Fine-Gray competing-risk regression with death as a competing risk. Bootstrap-corrected c-statistic for the reoperation model: 0.71.

The complications model showed adequate calibration (Hosmer-Lemeshow *χ*^2^ = 5.24, df = 8, *P* = 0.73) and acceptable discrimination (AUC = 0.74, 95% CI: 0.66–0.82).

For the specific outcome of reoperation, given the limited number of events (*n* = 25) and the potential for overfitting, competing-risk regression (Fine-Gray model) identified three independent predictors of higher cumulative reoperation incidence: PFNA procedure (compared to HA) (sHR = 2.78, 95% CI: 1.05–7.36, *P* = 0.04), AO/OTA type A3 fracture (sHR = 2.51, 95% CI: 1.02–6.18, *P* = 0.045), and osteoporosis (*T*-score ≤ −2.5) (sHR = 2.84, 95% CI: 1.48–5.45, *P* = 0.002) (Table 4, complete regression details are shown in Supplementary Table S3). Bootstrap internal validation with 1,000 resamples yielded a bias-corrected c-statistic of 0.71, indicating acceptable discrimination and no substantial overfitting.

Kaplan–Meier analysis with reoperation as the endpoint demonstrated better reoperation-free survival in the HA group compared to the PFNA group (log-rank *P* = 0.03) (Figure 2). When death was treated as a competing event using the Fine-Gray model, PFNA remained associated with a higher cumulative incidence of reoperation (subdistribution HR = 2.78, 95% CI: 1.05–7.36, *P* = 0.04) (Table 4). The corresponding cumulative incidence estimates are shown in Table 5.

**Figure 2:**
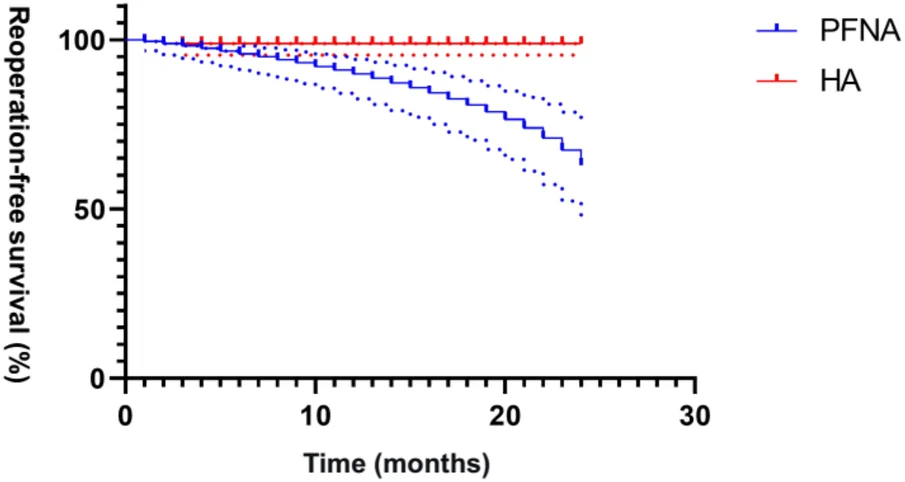
Kaplan–Meier curves for reoperation-free survival. Blue line: PFNA group (*n* = 96); red line: HA group (*n* = 78). Tick marks indicate censored observations. Log-rank *P* = 0.03. Numbers at risk at 0, 6, 12, 18, and 24 months: PFNA: 96, 92, 85, 82, 77; HA: 78, 76, 74, 72, 72.

**Table 5:** Cumulative incidence of reoperation with death as competing risk.

Time point	PFNA group (*n* = 96)	HA group (*n* = 78)	*P* value*
12 months	12.5% (95% CI: 7.3–19.2)	5.1% (95% CI: 2.0–11.8)	0.04
24 months	19.8% (95% CI: 13.1–28.5)	7.7% (95% CI: 3.4–15.6)	0.028

* Fine-Gray test.

To assess the potential impact of loss to follow-up on our primary outcomes, we performed a best-case/worst-case sensitivity analysis for mortality and reoperation. In the best-case scenario, the 2-year mortality rate was 9.8% in the PFNA group and 6.6% in the HA group (*P* = 0.09). In the worst-case scenario, the rates were 14.3% and 3.3%, respectively (*P* = 0.01). Although statistical significance varied across scenarios, the direction of the mortality difference remained consistent. For reoperation, the between-group difference remained significant across all sensitivity scenarios. These results suggest that the main findings are robust to assumptions about loss to follow-up (Supplementary Table S4).

## 4 Discussion

This retrospective comparative study investigated the clinical outcomes of PFNA internal fixation vs. uncemented bipolar hemiarthroplasty with long femoral stem in elderly patients with unstable extracapsular proximal femur fractures (AO/OTA types A2 and A3). Our findings demonstrate that although PFNA was associated with a shorter operative time (59.6 vs. 74.2 min, *P* < 0.001), hemiarthroplasty with a long stem provides superior early functional recovery and significantly lower reoperation rates and mortality. These results have crucial implications for surgical decision-making, although causal conclusions cannot be drawn due to the non-randomized design.

### 4.1 Internal fixation failure in unstable fracture patterns

Our finding of a 19.8% reoperation rate, with complications including internal fixation failure and nonunion, and the Kaplan–Meier survival analysis also demonstrating significantly worse results in the PFNA group, are consistent with previous reports in the literature. Failure rates for internal fixation of unstable peri-trochanteric fractures have been reported to range from 6% to 20% (21). A 2025 meta-analysis comparing cementless bipolar hemiarthroplasty (CBHA) and PFNA in unstable intertrochanteric fractures found that CBHA offers shorter weight-bearing duration and reduced prosthetic loosening, though with greater blood loss and longer operative times (15). Zhang et al. reported that among 96 intertrochanteric fractures treated with internal fixation, AO/OTA type A3 fractures had the highest failure rate, and patients undergoing conversion to arthroplasty had a significantly higher mean age (72.13 ± 13.00 years) than those receiving revision internal fixation (14).

The high failure rate of internal fixation in AO/OTA type A2 and A3 fractures can be attributed to the biomechanical instability inherent in these fracture patterns (14, 15, 21). Type A2 fractures are characterized by multi-fragmentary involvement with disruption of the posteromedial cortical support, which serves as the primary load-bearing structure in the proximal femur (14, 21). Type A3 fractures, with reverse obliquity or transverse patterns, are even more challenging because the fracture line orientation creates a lateralizing force that promotes medial displacement and implant failure (15). These unstable factors inevitably increase the risk of internal fixation failure and reoperation rate.

### 4.2 Osteoporosis impact on internal fixation outcomes

Osteoporosis emerged as another critical factor influencing internal fixation outcomes in our study. The prevalence of osteoporosis was high in both groups (PFNA: 84.4%, HA: 87.2%), reflecting the typical bone quality of the elderly hip fracture population (6). In patients with severe osteoporosis (*T*-score ≤ −2.5), the PFNA group had a significantly higher complication rate (48.8%) compared to the HA group (30.8%, *P* = 0.04). Multivariable analysis confirmed osteoporosis as an independent risk factor for overall complications (OR = 2.18, 95% CI: 1.12–4.24).

A previous study found that patients with displaced femoral neck fractures treated with internal screw fixation, 43.4% suffered from fixation failure, and 81.0% of those had osteoporosis (22). Multivariate analysis demonstrated that osteoporosis was strongly associated with treatment failure (OR = 2.79, 95% CI: 1.26–6.15, *P* = 0.01), leading the authors to conclude that patients with osteoporosis should not be offered internal fixation after hip fracture (21). A systematic review by Goldhahn et al. similarly concluded that osteoporotic bone provides inferior purchase for implants, leading to higher rates of mechanical failure, particularly in unstable fracture configurations (6). Bone mineral density cut-offs for predicting cut-out risk have also been established, with each 1 SD decrease increasing risk by approximately 30% (6, 10).

### 4.3 The immobilization challenge and its consequences

Perhaps the most clinically significant disadvantage of internal fixation in our study was the prolonged period of protected weight-bearing required. PFNA patients remained non-weight-bearing or toe-touch weight-bearing for a mean of 62.8 days, compared to only 4.2 days in the HA group (*P* < 0.001). This extended period of restricted mobility in an already vulnerable geriatric population has profound implications for patient outcomes, and significantly increases the risk of DVT (8, 9), which is consistent with our result (14.6% vs. 3.8%, *P* = 0.02).

A 2022 study by Song et al. comparing PFNA and bipolar hemiarthroplasty in unstable comminuted intertrochanteric fractures similarly found that hemiarthroplasty allows earlier return to weight-bearing activity (23). Koval et al. demonstrated that postoperative weight-bearing restrictions after hip fracture surgery are associated with increased complications and delayed functional recovery (24). Horton et al. reported that delayed mobilization increases the risk of perioperative complications including deep vein thrombosis, pulmonary embolism, pneumonia, and pressure ulcers (25). In our study, although the differences in individual medical complications did not reach statistical significance except for DVT, there was a consistent trend toward higher rates of pneumonia (8.3% vs. 6.4%) and pressure ulcers (6.3% vs. 2.6%) in the PFNA group. The higher incidence of DVT and the cumulative burden of these immobilization-related complications contribute to the substantial morbidity and mortality observed after hip fractures (26).

The inability to bear weight early after internal fixation also has functional consequences. The HA group achieved significantly better HHS at 3 and 6 months postoperatively, and significantly higher Barthel Index scores at 12 and 24 months, indicating better activities of daily living performance. This early functional advantage is clinically significant, as it may reduce the need for institutional care and facilitate return to pre-injury living arrangements. Although functional outcomes converged by 12–24 months, the quality of life during the first year after fracture-a critical period for elderly patients-was superior in the HA group.

### 4.4 Hemiarthroplasty in unstable fractures and its biomechanical rationale

Our results support hemiarthroplasty for elderly patients with unstable extracapsular proximal femur fractures, demonstrating significantly lower reoperation rates, lower mortality, and earlier functional recovery in the HA group. A 2025 meta-analysis by Mohamed et al. including 240 patients concluded that CBHA offers shorter weight-bearing duration and reduced prosthetic loosening compared to PFNA, with comparable functional recovery and hospital stay (15).

Despite the advantages of hemiarthroplasty demonstrated above, its biomechanical challenges in treating unstable extracapsular fractures merit further discussion. The use of a long femoral stem in hemiarthroplasty for extracapsular proximal femur fractures addresses several biomechanical challenges unique to this fracture type. By bypassing the fracture site and achieving fixation in the intact diaphysis, long stems create a load-sharing construct that protects the reconstructed proximal femur during weight-bearing (27). This is especially important in osteoporotic bone, where proximal fixation alone may be insufficient to resist the deforming forces of weight-bearing and muscle contraction. A 2022 study by Cai et al. found that while cementless bipolar hemiarthroplasty showed no obvious perioperative advantage over PFNA in elderly osteoporotic patients, the joint replacement allowed for earlier ambulation and rapid recovery of hip joint function (28).

Cementless hemiarthroplasty has historically been associated with a risk of periprosthetic fracture, recent high-level evidence indicates the relationship between cementless fixation and periprosthetic fracture risk is not a direct causal one. A 2025 meta-analysis demonstrated that overall periprosthetic fracture rates were comparable between cementless and cemented fixation (OR = 1.38, 95% CI: 0.95–2.02, *P* = 0.089) (29). Furthermore, concerns regarding bone cement implantation syndrome (BCIS)-characterized by hypoxia, hypotension and cardiovascular compromise-supported our preference for cementless fixation in elderly patients with cardiovascular comorbidities (30). Our favorable outcomes, including early weight-bearing tolerance and the absence of periprosthetic fractures in this cohort, support the clinical feasibility of this approach in selected patients.

In addition, inadequate reconstruction of greater trochanter can shorten the abductor moment arm, leading to Trendelenburg gait, reduced walking endurance, and increased prosthetic dislocation risk due to insufficient soft tissue tension (31). Furthermore, trochanteric nonunion, reported in up to 10%–15% of cases, may necessitate revision surgery and compromise long-term functional outcomes. To mitigate these risks, we performed meticulous anatomic reduction and fixation of the greater trochanter using cerclage wires or cables in all HA patients, combined with the use of a long femoral stem for distal diaphyseal bypass fixation, represents the biomechanical rationale underlying our surgical strategy for this challenging patient population (32).

### 4.5 Functional outcomes

A 2025 meta-analysis by the Russian Ilizarov Scientific Center, including six cohort studies with 495 participants, found that bipolar hemiarthroplasty had significantly better Harris Hip Scores at 1 and 3 months postoperatively (*P* < 0.00001 and *P* = 0.001, respectively) and enabled earlier weight-bearing (*P* = 0.003) compared to PFNA (12). At 6 and 12 months, the differences were no longer statistically significant, which is consistent with our finding that functional outcomes converged by 12–24 months. The meta-analysis by Kumar et al. comparing proximal femoral nailing vs. hemiarthroplasty for AO/OTA A2 and A3 fractures similarly demonstrated that hemiarthroplasty was associated with earlier mobilization and lower reoperation rates (33).

### 4.6 Mortality

The 2-year mortality rate was observed to be lower in the HA group than in the PFNA group (2.6% vs. 11.5%, *P* = 0.02). A 2019 study by Zhou et al. comparing PFNA and cementless bipolar hemiarthroplasty in elderly intertrochanteric fracture patients found that both are safe and effective treatments, but hemiarthroplasty allows faster mobilization and recovery (34). A 2017 study by Esen et al. evaluating PFNA vs. cemented bipolar hemiarthroplasty found favorable outcomes for the arthroplasty group in terms of early mortality (35).

However, a 2020 meta-analysis by Tu et al. found no significant difference in 1- or 2-year mortality between internal fixation and hemiarthroplasty groups (13). Thus, our observation may contrast with some existing literature, and these differences warrant special consideration. The characteristics of our patients (mean age 81 and 83 years, high prevalence of osteoporosis at 84%–87%, and significant comorbidity burden with mean CCI of 5.3–5.5) may represent a distinct subgroup where hemiarthroplasty confers survival benefits not seen in other populations. The use of long femoral stems in our HA group provides distal diaphyseal fixation beyond the fracture site, enabling significantly earlier ambulation (2.8 vs. 6.2 days) and immediate full weight-bearing, reducing immobilization-related complications such as pneumonia, pressure ulcers, DVT and thromboembolic events. Moreover, the significantly lower reoperation rate in the HA group (7.7% vs. 14.6%, *P* = 0.04) may have contributed to improved survival, as each reoperation exposes frail elderly patients to additional surgical stress and complications. However, given the non-randomized design of this study with only 13 death events at 2 years and the potential for residual confounding by unmeasured factors, the statistical power is insufficient for any meaningful adjusted survival analysis and this finding should be interpreted with caution. It is possible that this difference reflects, at least in part, baseline prognostic imbalances rather than a direct effect of the surgical procedure itself.

### 4.7 Strengths of the study

Despite these limitations, our study has several notable strengths. First, we focused specifically on unstable fracture patterns (AO/OTA types A2 and A3), excluding stable fractures that have favorable outcomes with internal fixation. This narrow focus enhances the clinical relevance of our findings for the challenging patient population where treatment decisions are most difficult. Second, we conducted comprehensive assessments including detailed comorbidity indices, cognitive function testing, and fracture characteristics, ensuring that the groups were well-matched and allowing for robust multivariable adjustment. The inclusion of cognitive function assessment is particularly important, as cognitive impairment is common in this population and may influence both treatment selection and outcomes. Third, we specifically examined the role of long-stem hemiarthroplasty, providing evidence for this technical refinement that is increasingly used in clinical practice but has limited supporting data.

### 4.8 Study limitations

Several limitations of this study should be acknowledged when interpreting the results. First, this was a retrospective non-randomized design study with inherent limitations including selection bias, information bias, and confounding by indication. However, it should be noted that the two groups demonstrated excellent comparability across all demographic and clinical characteristics, suggesting that the allocation process achieved a reasonable balance between groups. Moreover, multivariable logistic regression analysis, which adjusted for potential confounders, consistently identified PFNA as an independent risk factor for complications and reoperations, suggesting that the observed differences are unlikely to be entirely attributable to baseline imbalances. However, the number of death events in this study was limited, which precluded any meaningful adjusted survival analysis (Cox regression) and significantly reduced the statistical power to detect or refute mortality differences. And detailed technical parameters and interobserver reliability analysis for radiographic assessments were not systematically recorded in this retrospective dataset, their absence precludes adjustment for technical confounding. Therefore, residual confounding from unmeasured variables cannot be completely excluded, this observed association and mortality findings should be interpreted with caution and cannot be construed as causal, which should be confirmed by future prospective randomized trials. Second, the study was conducted at a single institution, which may limit generalizability to other settings with different patient populations, surgical techniques, and rehabilitation protocols. However, the single-center design also ensured standardization of surgical techniques and perioperative care, reducing variability that might confound multicenter studies. Third, the follow-up period of 24 months, while adequate to capture most complications and reoperations, may not detect very late failures such as aseptic loosening in the HA group or late implant breakage in the PFNA group. Longer follow-up studies are needed to assess the durability of both procedures. Fourth, we did not perform systematic screening for deep vein thrombosis in asymptomatic patients, potentially underestimating the true incidence of thromboembolic events. However, clinically significant events were captured, and the comparison between groups remains valid. Fifth, the two groups were managed with fundamentally different postoperative weight-bearing protocols, reflecting distinct rehabilitation pathways for internal fixation vs. arthroplasty. This rehabilitation bias represents a significant source of confounding inherent to the comparative effectiveness design, and our findings should be interpreted with this limitation in mind. Sixth, although we employed competing-risk regression and bootstrap validation to mitigate overfitting, the limited number of reoperation events (*n* = 25) restricted the number of covariates that could be included in the final model. The events-per-variable ratio was approximately 8:1, which, while improved from the initial model, remains below the ideal threshold of 10:1. Therefore, the reoperation findings should be interpreted with caution and warrant external validation in larger prospective cohorts. Additionally, although the loss-to-follow-up rate was similar between groups (PFNA: 17.7%; HA: 17.9%) and sensitivity analyzes supported the robustness of our main findings, attrition bias cannot be completely excluded. Patients lost to follow-up may differ systematically from those retained (e.g., in functional status or socioeconomic factors), potentially affecting the generalizability of our results.

## 5 Conclusions

As the global population continues to age and the incidence of extracapsular proximal femur fractures rises, the need for evidence-based treatment algorithms becomes increasingly urgent. This study contributes to the growing body of evidence supporting the selective use of long-stem hemiarthroplasty for unstable extracapsular proximal femur fractures in appropriately selected elderly patients. However, these findings should not be interpreted as causal evidence, prospective randomized controlled trials are needed to confirm these observed advantages and to establish definitive treatment recommendations for this challenging patient population.

## Data Availability

The raw data supporting the conclusions of this article will be made available by the authors, without undue reservation.

## References

[1] Johnell O Kanis JA. An estimate of the worldwide prevalence and disability associated with osteoporotic fractures. Osteoporos Int. (2006) 17(12):1726–33. 10.1007/s00198-006-0172-4 16983459

[2] Veronese N Maggi S. Epidemiology and social costs of hip fracture. Injury. (2018) 49(8):1458–60. 10.1016/j.injury.2018.04.015 29699731

[3] Haidukewych GJ. Intertrochanteric fractures: ten tips to improve results. Instr Course Lect. (2010) 59:503–9. 20415401

[4] He S Yan B Zhu J Huang X Zhao J. High failure rate of proximal femoral locking plates in fixation of trochanteric fractures. J Orthop Surg Res. (2018) 13(1):248. 10.1186/s13018-018-0951-6 30290848 PMC6173843

[5] Palm H Lysén C Krasheninnikoff M Holck K Jacobsen S Gebuhr P. Intramedullary nailing appears to be superior in pertrochanteric hip fractures with a detached greater trochanter: 311 consecutive patients followed for 1 year. Acta Orthop. (2011) 82(2):166–70. 10.3109/17453674.2011.566143 21434790 PMC3235286

[6] Goldhahn J Seebeck J Frei R Frenz B Antoniadis I Schneider E. New implant designs for fracture fixation in osteoporotic bone. Osteoporos Int. (2005) 16(Suppl 2):S112–9. 10.1007/s00198-004-1765-4 15536537

[7] NIH Consensus Development Panel on Osteoporosis Prevention, Diagnosis, and Therapy. Osteoporosis prevention, diagnosis, and therapy. J Am Med Assoc. (2001) 285(6):785–95. 10.1001/jama.285.6.785 11176917

[8] Boddaert J Cohen-Bittan J Khiami F Le Manach Y Raux M Beinis JY. Postoperative admission to a dedicated geriatric unit decreases mortality in elderly patients with hip fracture. PLoS One. (2014) 9(1):e83795. 10.1371/journal.pone.0083795 24454708 PMC3893076

[9] Abrahamsen B Van Staa T Ariely R Olson M Cooper C. Excess mortality following hip fracture: a systematic epidemiological review. Osteoporos Int. (2009) 20(10):1633–50. 10.1007/s00198-009-0920-3 19421703

[10] Tajima K Yoshida M Murakami D Nishimura T Hirakawa A Uenishi N. Primary bipolar hemiarthroplasty as a treatment option for unstable intertrochanteric fractures. Fujita Med J. (2020) 6(4):122–7. 10.20407/fmj.2019-022 35111533 PMC8761824

[11] Hongku N Woratanarat P Nitiwarangkul L Rattanasiri S Thakkinstian A. Fracture fixation versus hemiarthroplasty for unstable intertrochanteric fractures in elderly patients: a systematic review and network meta-analysis of randomized controlled trials. Orthop Traumatol Surg Res. (2022) 108(1):102838. 10.1016/j.otsr.2021.102838 33529729

[12] Damara IGAD Wijaya NSN Dusak IWS. Proximal femoral nail antirotation versus bipolar hemiarthroplasty for intertrochanteric fractures: a meta-analysis. Genij Ortop. (2025) 31(2):252–62. 10.18019/1028-4427-2025-31-2-252-262

[13] Tu DP Liu Z Yu YK Xu C Shi XL. Internal fixation versus hemiarthroplasty in the treatment of unstable intertrochanteric fractures in the elderly: a systematic review and meta-analysis. Orthop Surg. (2020) 12(4):1053–64. 10.1111/os.12736 32691520 PMC7454150

[14] Zhang Y Yang F Yu C Zhu H Chen E Li H. Clinical outcomes and revision strategies following failed internal fixation of peri-trochanteric femoral fractures: a retrospective cohort study. Front Med. (2026) 13:1773816. 10.3389/fmed.2026.1773816 PMC12996186 41859146

[15] Yousif Mohamed AM Salih M Mohamed M Abbas AE Elsiddig M Osama Omar M. Comparative efficacy of cementless bipolar hemiarthroplasty and proximal femoral nail anti-rotation in unstable intertrochanteric fractures: a meta-analysis. World J Orthop. (2025) 16(7):107950. 10.5312/wjo.v16.i7.107950 40698275 PMC12278281

[16] Bonnaire F Weber A Bösl O Eckhardt C Schwieger K Linke B. “Cutting out” bei pertrochantären Frakturen – ein Problem der Osteoporose? [“Cutting out” in pertrochanteric fractures–problem of osteoporosis?]. Unfallchirurg. (2007) 110(5):425–32, German. 10.1007/s00113-007-1248-0 17361444

[17] Kuzyk PR Lobo J Wunder JS Schemitsch EH. The biomechanics of fixed-angle bone plates. J Bone Joint Surg Am. (2011) 93(19):1865–74. 10.2106/JBJS.J.0173922012523

[18] Borders C Sajjadi SA. Diagnosis and management of cognitive concerns in the oldest-old. Curr Treat Options Neurol. (2021) 23(3):10. 10.1007/s11940-021-00665-5 33786000 PMC7994350

[19] Liu L Deng Y Qin Z Zhang Y Yang X Feng J. Clinical prediction model and 2-year mortality for multiple organ dysfunction in patients aged 80 years or older following hip fracture surgery: a prospective cohort study. Front Med. (2025) 25(12):1515557. 10.3389/fmed.2025.1515557 PMC12331632 40786094

[20] Dimai HP. Use of dual-energy x-ray absorptiometry (DXA) for diagnosis and fracture risk assessment; WHO-criteria, T- and Z-score, and reference databases. Bone. (2017) 104:39–43. 10.1016/j.bone.2016.12.016 28041872

[21] Cui K Cao YQ Tai PY. Risk factors analysis on failure in intramedullary fixation for intertrochanteric fracture. Chin J Gen Pract. (2016) 14(4):566–8. 10.16766/j.cnki.issn.1674-4152.2016.04.017

[22] Bhandari M Devereaux PJ Swiontkowski MF Tornetta P 3rd Obremskey W Koval KJ. Internal fixation compared with arthroplasty for displaced fractures of the femoral neck. A meta-analysis. J Bone Joint Surg Am. (2003) 85(9):1673–81. 10.2106/00004623-200309000-00004 12954824

[23] Song QC Dang SJ Zhao Y Wei L Duan DP Wei WB. Comparison of clinical outcomes with proximal femoral nail anti-rotation versus bipolar hemiarthroplasty for the treatment of elderly unstable comminuted intertrochanteric fractures. BMC Musculoskelet Disord. (2022) 23(1):628. 10.1186/s12891-022-05583-4 35778710 PMC9248155

[24] Koval KJ Cooley MR Zuckerman JD. Postoperative weight-bearing restrictions after hip fracture surgery: effect on complications and functional recovery. J Orthop Trauma. (2008) 22(5):321–5. 10.1097/BOT.0b013e31817d9774

[25] Horton I Bourget-Murray J Buth O Backman C Green M Papp S. Delayed mobilization following admission for hip fracture is associated with increased morbidity and length of hospital stay. Can J Surg. (2023) 66(4):E432–8. 10.1503/cjs.006822 37643796 PMC10473868

[26] Xia ZH Chen WH Wang Q. Risk factors for venous thromboembolism following surgical treatment of fractures: a systematic review and meta-analysis. Int Wound J. (2023) 20(4):995–1007. 10.1111/iwj.13949 36382679 PMC10030940

[27] Hennicke NS Kluess D Sander M. Influence of stem design parameters on periprosthetic femoral fractures examined by subject specific finite element analyses. Med Eng Phys. (2023) 119:104032. 10.1016/j.medengphy.2023.104032 37634909

[28] Cai C Tian L Chen Z Sun P Wang G. Cementless bipolar hemiarthroplasty compared with proximal femoral nail anti-rotation of unstable intertrochanteric fractures in senile patients with osteoporosis: a retrospective study. BMC Musculoskelet Disord. (2022) 23(1):461. 10.1186/s12891-022-05426-2 35578265 PMC9112522

[29] Borazjani R Ayatizadeh SH Sattarian M Moein SA Mont MA Kreuzer SW. Quadrangular taper cementless designs are associated with lower risks of postoperative periprosthetic fracture compared with cemented stems and other cementless designs following primary total hip arthroplasty: a systematic review and meta-analysis. J Arthroplasty. (2026) 12:S0883-5403(26)00031-8. 10.1016/j.arth.2026.01.031 41534614

[30] Al-Husinat L Jouryyeh B Al Sharie S Al Modanat Z Jurieh A Al Hseinat L. Bone cement and its anesthetic complications: a narrative review. J Clin Med. (2023) 12(6):2105. 10.3390/jcm12062105 36983108 PMC10056839

[31] Heller S Kirschner S Günther KP Lützner J. Die Rekonstruktion des Trochanter major mit einem Kabelsystem bei der Hüft-TEP-Implantation [Greater trochanteric reconstruction with cable system in hemiarthroplasty]. Z Orthop Unfall. (2015) 153(1):57–62, German. 10.1055/s-0034-1396148

[32] Karaali E Çiloğlu O. Metaphyseal vs. diaphyseal fixed-stem hemiarthroplasty in treating unstable intertrochanteric fractures in elderly patients. Ulus Travma Acil Cerrahi Derg. (2021) 27(1):104–8. 10.14744/tjtes.2020.09990 33394460

[33] Kumar P Rajnish RK Sharma S Dhillon MS. Proximal femoral nailing is superior to hemiarthroplasty in AO/OTA A2 and A3 intertrochanteric femur fractures in the elderly: a systematic literature review and meta-analysis. Int Orthop. (2020) 44(4):623–33. 10.1007/s00264-019-04351-9 31201487

[34] Zhou S Liu J Zhen P Shen W Chang Y Zhang H. Proximal femoral nail anti-rotation versus cementless bipolar hemiarthroplasty for unstable femoral intertrochanteric fracture in the elderly: a retrospective study. BMC Musculoskelet Disord. (2019) 20(1):500. 10.1186/s12891-019-2793-8 31664982 PMC6820901

[35] Esen E Dur H Ataoğlu MB Ayanoğlu T Turanlı S. Evaluation of proximal femoral nail-antirotation and cemented, bipolar hemiarthroplasty with calcar replacement in treatment of intertrochanteric femoral fractures in terms of mortality and morbidity ratios. Eklem Hastalik Cerrahisi. (2017) 28(1):35–40. 10.5606/ehc.2017.53247 28291437

