# Supplementary material for: Clinical outcomes of PFNA vs. long-stem hemiarthroplasty for unstable proximal femur fractures in older adults: a retrospective cohort study
Source: Front Surg. 2026 Oct 5;13:1889099. doi: 10.3389/fsurg.2026.1889099 (PMC13677882; doi:10.3389/fsurg.2026.1889099)
Supplement: Supplementary file 1 [file Table1.docx]

**Table S1. Demographic and Clinical Characteristics of Patients (Overall Comorbidities)**

| Characteristic | PFNA Group (n=96) | HA Group (n=78) | P-value |
| --- | --- | --- | --- |
| Comorbidities (n, %) |  |  |  |
| Hypertension | 68 (70.8%) | 57 (73.1%) | 0.74‡ |
| Diabetes mellitus | 41 (42.7%) | 32 (41.0%) | 0.82‡ |
| Coronary artery disease | 33 (34.4%) | 28 (35.9%) | 0.83‡ |
| Heart failure | 19 (19.8%) | 17 (21.8%) | 0.75‡ |
| Chronic atrial fibrillation | 15 (15.6%) | 14 (17.9%) | 0.68‡ |
| Chronic obstructive pulmonary disease | 22 (22.9%) | 17 (21.8%) | 0.86‡ |
| Chronic kidney disease (Stage ≥3) | 13 (13.5%) | 12 (15.4%) | 0.73‡ |
| Cerebrovascular disease (stroke/TIA) | 18 (18.8%) | 16 (20.5%) | 0.77‡ |
| Peripheral vascular disease | 10 (10.4%) | 7 (9.0%) | 0.75‡ |
| Rheumatoid arthritis | 7 (7.3%) | 5 (6.4%) | 0.82‡ |
| Medication History (n, %) |  |  |  |
| Antiplatelet/Anticoagulant use | 47 (49.0%) | 41 (52.6%) | 0.64‡ |
| - Aspirin | 34 (35.4%) | 29 (37.2%) | 0.81‡ |
| - Clopidogrel | 10 (10.4%) | 9 (11.5%) | 0.81‡ |
| - Warfarin/DOACs | 3 (3.1%) | 3 (3.8%) | 1.00§ |
| Statin use | 43 (44.8%) | 34 (43.6%) | 0.87‡ |
| Oral corticosteroid use | 8 (8.3%) | 5 (6.4%) | 0.63‡ |
| Preoperative hemoglobin (g/dL) | 11.7 ± 1.7 | 11.5 ± 1.9 | 0.46† |
| Cognitive Function Assessment |  |  |  |
| MMSE score (mean ± SD) | 23.8 ± 4.3 | 23.4 ± 4.6 | 0.55† |
| Cognitive status (n, %) |  |  | 0.62‡ |
| - Normal (MMSE ≥25) | 52 (54.2%) | 40 (51.3%) |  |
| - Mild impairment (MMSE 21-24) | 33 (34.4%) | 28 (35.9%) |  |
| - Moderate impairment (MMSE 10-20) | 11 (11.5%) | 10 (12.8%) |  |
| Preoperative delirium (CAM positive) (n, %) | 7 (7.3%) | 5 (6.4%) | 0.82‡ |
| Preoperative Barthel Index (mean ± SD) | 81.6 ± 16.2 | 79.8 ± 17.8 | 0.49† |

† Independent samples t-test; ‡ Chi-square test; § Fisher's exact test
Abbreviations: PFNA, proximal femoral nail antirotation; HA, hemiarthroplasty; SD, standard deviation; TIA, transient ischemic attack; DOACs, direct oral anticoagulants; MMSE, Mini-Mental State Examination; CAM, Confusion Assessment Method

**Table S2. Multivariable Logistic Regression Analysis for Overall Complications**

| **Variable** | **Coefficient (β)** | **SE** | **Wald χ²** | **OR** | **95% CI** | **P** |
| --- | --- | --- | --- | --- | --- | --- |
| Age ≥85 years | 1.044 | 0.391 | 7.13 | 2.84 | 1.32–6.11 | 0.008 |
| CCI >5 | 0.941 | 0.354 | 7.07 | 2.56 | 1.28–5.12 | 0.008 |
| Osteoporosis (T-score ≤ -2.5) | 0.779 | 0.34 | 5.25 | 2.18 | 1.12–4.24 | 0.02 |
| AO/OTA type A3 | 0.673 | 0.333 | 4.08 | 1.96 | 1.02–3.77 | 0.04 |
| PFNA (vs. HA) | 0.652 | 0.299 | 4.76 | 1.92 | 1.35–2.73 | 0.01 |

Hosmer-Lemeshow goodness-of-fit: χ² = 5.24, df = 8, P = 0.73; AUC = 0.74 (95% CI: 0.66–0.82). VIF < 2.0 for all variables, indicating no significant collinearity.

**Table S3. Reoperation（Fine-Gray model）**

| Variable | β | SE | Wald χ² | sHR | 95% CI | P |
| --- | --- | --- | --- | --- | --- | --- |
| PFNA (vs. HA) | 1.022 | 0.497 | 4.23 | 2.78 | 1.05–7.36 | 0.04 |
| AO/OTA type A3 | 0.921 | 0.459 | 4.02 | 2.51 | 1.02–6.18 | 0.045 |
| Osteoporosis | 1.044 | 0.332 | 9.89 | 2.84 | 1.48–5.45 | 0.002 |

Bootstrap-corrected c-statistic: 0.71.

**Table S4. Sensitivity Analysis for Loss to Follow-up**

| Outcome | Scenario | PFNA Group (n=96) | HA Group (n=78) | P Value* |
| --- | --- | --- | --- | --- |
| 2-year mortality | Main analysis (observed) | 11 (11.5%) | 2 (2.6%) | 0.02 |
|  | Best-case scenario† | 9.8% | 6.6% | 0.09 |
|  | Worst-case scenario‡ | 14.3% | 3.3% | 0.01 |
| Reoperation | Main analysis (observed) | 19 (19.8%) | 6 (7.7%) | 0.028 |
|  | Best-case scenario† | 17.9% | 9.8% | 0.04 |
|  | Worst-case scenario‡ | 21.7% | 5.6% | 0.02 |

*Fisher's exact test (two-sided).
†Best-case scenario: all patients lost to follow-up in the PFNA group assumed to have favorable outcomes (alive for mortality; no reoperation for reoperation); all those lost in the HA group assumed to have unfavorable outcomes (dead; reoperation).
‡Worst-case scenario: all patients lost to follow-up in the PFNA group assumed to have unfavorable outcomes (dead; reoperation); all those lost in the HA group assumed to have favorable outcomes (alive; no reoperation).
PFNA, proximal femoral nail antirotation; HA, hemiarthroplasty.

**Table S5. Number of Patients Assessed for HHS at Each Time Point**

| Time Point | PFNA Group (n=96) | HA Group (n=78) |
| --- | --- | --- |
| Preoperative | 96 (100%) | 78 (100%) |
| 3 months | 88 (91.7%) | 72 (92.3%) |
| 6 months | 84 (87.5%) | 70 (89.7%) |
| 12 months | 80 (83.3%) | 68 (87.2%) |
| 24 months | 74 (77.1%) | 64 (82.1%) |
